# Hyperparasitaemia during clinical malaria episodes in infants aged 0–24 months and its association with in utero exposure to *Plasmodium falciparum*

**DOI:** 10.1186/s13104-018-3339-0

**Published:** 2018-04-04

**Authors:** Boniphace Sylvester, Dinah B. Gasarasi, Said Aboud, Donath Tarimo, Siriel Massawe, Rose Mpembeni, Gote Swedberg

**Affiliations:** 1 Department of Parasitology and Medical Entomology, School of Public Health and Social Sciences, Muhimbili University of Health and Allied Sciences, P.O.BOX 65001, Dar es Salaam, Tanzania; 2 Department of Microbiology and Immunology, School of Medicine, Muhimbili University of Health and Allied Sciences, P.O.BOX 65001, Dar es Salaam, Tanzania; 3 Department of Obstetrics and Gynaecology, School of Medicine, Muhimbili University of Health and Allied Sciences, P.O.BOX 65001, Dar es Salaam, Tanzania; 4 Department of Epidemiology and Biostatistics, School of Public Health and Social Sciences, Muhimbili University of Health and Allied Sciences, Dar es Salaam, Tanzania; 5 Department of Medical Biochemistry and Microbiology, Biomedical Centre, Uppsala University, P.O.BOX 582, Uppsala, Sweden

**Keywords:** Parasite density, *P. falciparum*, In utero, Malaria, Infants, Hyperparasitaemia

## Abstract

**Objective:**

Existing information has shown that infants who are prenatally exposed to *P. falciparum* are susceptible to subsequent malaria infections. However, the effect of prenatal exposure to *P. falciparum* on parasite density during clinical malaria episodes has not been fully elucidated. This study is a component of a prospective cohort study for which initial results have been published. This component was designed to determine the effect of prenatal exposure to *P. falciparum* on parasite density during clinical malaria episodes in the first 24 months of life. A total of 215 infants were involved and monitored for clinical malaria episodes defined by fever (≥ 37 °C) and parasitaemia. The geometric mean parasite counts between exposed and unexposed infants were compared using independent samples t test. The effect of in utero exposure to *P. falciparum* on parasite density was assessed using binary logistic regression.

**Results:**

The geometric mean parasite count per µl of blood during clinical malaria episodes in exposed infants was 24,889 (95% CI 18,286–31,490) while in unexposed infants it was 14,035 (95% CI 12,111–15,960), P < 0.05. Prenatal exposure to *P. falciparum* was associated with hyperparasitaemia during clinical malaria episodes (OR 7.04, 95% CI 2.31–21.74), while other factors were not significantly associated (P > 0.05).

## Introduction

*Plasmodium falciparum* malaria is a major cause of death in children in sub-Saharan Africa [1, 2]. In endemic regions, infants born to mothers with placental malaria are exposed in utero to *P. falciparum* antigens, like *P. falciparum* merozoite surface protein 1–19 (PfMSP1–19) and *P. falciparum* merozoite surface protein 2 (PfMSP2) [3–8]. Prenatal exposure to *P. falciparum* antigens affects the development of fetal immune cells and infant susceptibility to malaria infections [3–5]. Furthermore, prenatal exposure to *P. falciparum* is also associated with poor pregnancy outcomes [9, 10].

During childhood, on average children experience hyperparasitaemia in severe malaria [11]; however, hyperparasitaemia may be encountered both in severe and mild malaria infections [1]. This implies that severity of malaria disease may not only be attributable to high parasite density but also other factors like transmission season, use of long lasting insecticide treated bed nets (LLINs), parasite virulence and host immunity.

Although prenatal exposure has been demonstrated to influence susceptibility to malaria infection [12], the paucity of information on its effect on susceptibility to infection and disease severity requires further investigation. This study was designed to assess the effect of prenatal exposure to *P. falciparum* on parasite density during clinical malaria episodes in infants aged zero up to 24 months.

## Main text

### Methods

#### Study area

The detailed description of the study area was reported earlier [12]. Briefly, the study was conducted in Rufiji District Tanzania, characterized by perennial malaria transmission.

#### Study design and recruitment research participants

The study design, sample population, recruitment and follow up procedures were reported earlier [12]. Briefly, 215 infant mother pairs were recruited after parturition and placed into two categories, after establishing the mothers’ placental malaria status. Fifty mothers had placental malaria and their infants were categorized as exposed in utero to malaria and the remaining (n = 165) were unexposed. Recruited infants were followed from birth to 24 months, for 3 monthly visits. During visits, exposure to malaria infection was monitored, and clinical malaria episodes were diagnosed.

#### Diagnosis of placental malaria

Procedures for collection of placental tissues, storage and processing, for histological examinations were reported earlier [12]. Briefly, placental tissues were stored in 10% neutral buffered formalin, processed overnight on automatic processor, sectioned, mounted, stained using hematoxylin and eosin, dried and examined with a light microscope to establish placental malaria status.

#### Determination of parasite density

Capillary blood samples were collected at each visit for determination of parasite density and species identification. Thick and thin blood smears were prepared, the thin smears were fixed in methyl alcohol, dried, stained with 10% giemsa and examined using a light microscope at 100× objective for diagnosis and parasite quantification. Two laboratory technicians examined the slides, discrepancies in slide reading, were resolved by a third technologist and *P. falciparum* was the only parasite species identified. Parasite counts were expressed per µl of blood, assuming a leukocyte count of 8000 per µl. Levels of parasitaemia were categorized into low parasitaemia (< 1000 parasites/µl blood), intermediate parasitaemia (1000–9999 parasites/µl blood) and hyperparasitaemia (> 10,000 parasites/µl blood).

#### Determination of prenatal sensitization to *Plasmodium falciparum* antigens

Cord blood sera was collected following delivery and stored at − 20 °C at field site, then transported in ice parked cool box to Muhimbili University and transferred to – 80 °C freezers until use. Detailed procedures for determination of seropositivity and seronegativity for IgM against recombinant *P. falciparum* antigens (PfMSP1–19, PfMSP2) were described elsewhere [13]. Briefly, 100 μl of serum diluted to 1:1000 was added to duplicate wells and incubated overnight at 4 °C; washed and incubated with horseradish peroxidase conjugated goat anti-human IgM antibodies diluted at 1:6000 (Mabtech) for 3 h at room temperature. The test was developed with a substrate Tetramethylbenzidine at 4 °C for 10 min and reactions were stopped using 20 μl of 2 M H_2_SO_4_ per well. The optical density (OD) was measured at 450 nm. Control samples were used in each run and tested sera were defined as positive if they gave OD above the cut-off point (mean + 3SD) of negative control group born and living in Sweden. Based on the test results infants were categorized as, in utero sensitized (seropositivity for IgM) and non sensitized to *P. falciparum* antigens (seronegativity for IgM).

#### Data analysis

Data were analyzed using Statistical Package for Social Sciences (SPSS), IBM version 20. In order to use parametric tests data were log transformed. Assessment of effect of prenatal exposure to *P. falciparum* and other factors (live birth weight, gravidity, season of birth, IPTp-SP and use of LLINs) on hyperparasitaemia were assessed using binary logistic regression. The difference in the geometric mean parasite counts between exposed and unexposed was assessed using independent samples t test and the difference was judged at P < 0.05.

### Results

#### Socio-demographic characteristics of the study population

Detailed description of socio-demographic characteristics of the study population was reported earlier [12]. Briefly, the social demographic characteristics of the mothers were not statistically different. The social demographic characteristics of exposed and unexposed infants were not statistically different except birth weight which was statistically significant P < 0.05.

#### Parasite density during clinical malaria episodes in recruited infants and prenatal immunosensitization to PfMSP1–19 and PfMSP2

**Fig. 1:**
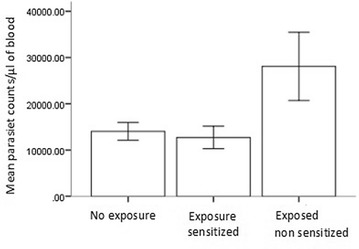
Mean parasite counts during clinical malaria episodes in exposed sensitized, exposed non sensitized and unexposed infants. The figure with standard error bars shows the geometric mean counts/µl of blood in the infants born to mothers without placental malaria (no exposure group), infants born to mother with placental malaria (pm+) and were seropositive for IgM against Pfmsp1–19 and Pfmsp2 in their cord blood sera (exposed sensitized group) and infants born to mothers with placental malaria (pm+) and were seronegative for IgM against Pfmsp1–19 and Pfmsp2 in their cord blood sera (exposed non-sensitized)

The geometric mean parasite count per µl of blood during clinical malaria episodes in exposed and unexposed infants were 24,889 (95% CI 18,286–31,490) 14,035 (95% CI 12,111–15,960) respectively and the difference was statistically significant (P < 0.05). Ninety percent (n = 45) of infants born to mothers with placental malaria were not sensitized to *P. falciparum* specific antigens while 10% (n = 5), had specific IgM for PfMSP1–19 and PfMSP2 in cord blood sera and were therefore sensitized to *P. falciparum* antigens. All infants born to mothers without placental malaria (unexposed) had no detectable IgM against *P. falciparum* antigens, mean parasite count during clinical malaria episodes in exposed sensitized infants was 12,731 (95% CI 9364–16,098) and in exposed-non sensitized it was 28,087 (95% CI 20,333–35,842) and the difference was statistically significant, P < 0.01 (Fig. 1).

#### Effect of prenatal exposure to *P. falciparum* on parasite density

Prenatal exposure to *P. falciparum* was significantly associated with hyperparasitaemia (P < 0.01, OR 7.04, 95% CI 2.31–21.74) during clinical malaria episodes in the first 2 years of life. Other factors; gravidity, season of birth, gender of infant, birth weight and IPT-SP were not significantly associated with hyperparasitaemia in a univariate analysis (Table 1); and these factors did not statistically influence the effect of prenatal exposure to *P. falciparum* on parasite density in a multivariate analysis.

**Table 1:** Effect of prenatal exposure to *P. falciparum*, gravidity, season of birth, birth weights and IPTp-SP on hyperparasitaemia: binary logistic regression (BLR)

Factors	OR (CI 95%)	P value
Non exposure to *P. falciparum*	1 (–)	–
Exposure to *P. falciparum*	7.04 (2.31–21.74)	< 0.01
Gravidity		
Primigravida	1 (–)	–
Multigravida	1.97 (0.717–5.125)	0.195
Season of birth		
Dry season	1 (–)	–
Wet season	0.994 (0.280–1.803)	0.992
Gender		
Males	1 (–)	–
Female	1.803 (0.673–4.831)	0.241
Birth weight		
(≥ 2.5 kg)	1 (–)	–
(≤ 2.5 kg)	2.267 (0.637–8.072)	0.207
IPTp-sp		
Use	1 (–)	–
Non use	0.711 (0.043–11.865)	0.812

The weight ≤ 2.5 kg was considered underweight while ≥ 2.5 was considered normal weight

*OR* odds ratio, *CI* confidence interval, *IPTp-SP* intermittent preventive treatment with sulfadoxine pyrimethamine. *BLR* binary logistic regression

### Discussion

Results of this study have shown that prenatal exposure to *P. falciparum* is significantly associated with hyperparasitaemia during clinical malaria episodes in the first 24 months of life in a malaria endemic area.

The findings of this study of hyperparasitaemia in infants prenatally exposed to *P. falciparum* may suggest inappropriate anti parasitic protective immunity. This suggestion is supported by observations from previous studies indicating that prenatal exposure to *P. falciparum* affected development of fetal regulatory and effector T cell responses [14, 15]. The effect of prenatal exposure to *P. falciparum* on fetal immune cells may persist to childhood; leading to inappropriate anti-parasitic immunity and failure of effective immune response to control parasite replication during clinical malaria episodes, with subsequent hyperparasitaemia. A study conducted in Kenya, demonstrated that prenatal exposure to *P. falciparum* affected the fetal immune cells and leads to immunosensitization or immunotolerance to malaria parasite antigens [4]. Although the present study did not directly assess the effect of prenatal exposure to *P. falciparum* on subsequent development of protective immunity; implicitly the findings of the current study corroborate the study in Kenya, in that 90% of the infants born to mothers with placental malaria were non sensitized to *P. falciparum* specific antigens (PfMSP1–19 and PfMSP2). In this study, a small proportion of 10% of the exposed infants were immunosensitized as demonstrated by IgM seropositivity, and had relatively lower parasitaemia compared to the 90% who were non sensitized. This observation indicates that early exposure to the parasite antigen may have implications on their capacity to mount appropriate immune response when naturally challenged with similar parasite antigens.

The vulnerability of exposed infants to high parasite density observed in this study is further corroborated by previous studies, which demonstrated that prenatal exposure to malaria may negatively affect the acquisition of antibodies against *P. falciparum* [5], rendering exposed infants more susceptible to malaria infection [5, 16–20].

Furthermore, the findings in this study, that prenatal exposure to *P. falciparum* predisposes the exposed infants to high parasite density during clinical malaria episodes, may be supported by observations from another study which indicated that; prenatal exposure to *P. falciparum* is associated with immaturity of fetal/neonatal antigen presenting cells and provide insufficient co-stimulatory signals to T cells which are important in combating parasitaemia [21].

In the pathogenesis of malaria infection, parasite density has been demonstrated to be associated with onset of severe malaria [22]. However, high parasite density has also been observed in infants with mild malaria infection. This implies that high parasite density may not be a sole indicator for the severity of malaria infection since other factors like gravidity; use of long lasting insecticide treated bed nets (LLINs) and season of birth may influence the severity of malaria and parasite density. Gravidity has been demonstrated to influence the acquisition of placental malaria, and primigravida mothers lack acquired antibodies against parasites binding chondroitin sulfate A that would protect them against placental malaria infection unlike multigravida mothers. Implicitly, infants born to primigravida mothers are more likely to be exposed to placental malaria which may subsequently influence their susceptibility to malaria infection. Similarly, the use of LLINs is likely to influence vector abundance and risk of malaria infection. Therefore, in situations where LLINs are not used, there may be increased exposure to mosquito bites and risk of malaria infection during high transmission season.

Hyperparasitaemia leads to destruction of host erythrocytes, consumption of host glucose, stimulation of lactate formation through hypoxia and alteration of erythrocyte membrane transport systems [23]. Thus infants exposed in utero to parasite antigens may be at higher risk of serious complications associated with malaria infection despite the fact that other factors may be at play in influencing levels of parasitaemia. However, the findings in this study of a high proportion of exposed non-sensitized infants (immunotolerant) may indicate that prenatal exposure to the parasite antigen plays a significant role in influencing hyperparasitaemia in malaria infection.

In this study, gravidity, use of LLINs, birth weight, age of mother at delivery, season of birth and use of IPTp-SP were not significantly associated with hyperparasitaemia during clinical malaria episodes. However, in another study conducted in Tanzania, season and use of LLINs were significantly associated with parasite density [1]. The observed inconsistencies in the role of other factors in influencing parasite density may be attributable to the differences in the study designs.

Prospectively, the findings of this study further emphasize the importance of preventing malaria in women of reproductive age in order to mitigate the exposure of the fetus to *P. falciparum* antigens.

### Conclusion

Prenatal exposure to *P. falciparum* is associated with hyperparasitaemia during clinical malaria episodes in the first 24 months of life.

## Limitation

Anti malaria campaigns were going on in the study area.

## Abbreviations

OR: odds ratio
BLR: binary logistic regression
BSA: bovine serum albumin
CI: confidence interval
ELISA: enzyme linked immunosorbant assay
GST: glutathione s-transferase
LLINs: long lasting Insecticide treated bed nets
PfMSP1: *Plasmodium falciparum* merozoite surface protein 1
PfMSP2: *Plasmodium falciparum* merozoite surface protein 2
PBS: phosphate buffer saline
pm+: placental malaria positive
pm−: placental malaria negative
TMB: tetra methyl benzidine
